# Changes During Reopening in Premature Constriction or Closure of the Ductus Arteriosus: A Report of Two Cases

**DOI:** 10.7759/cureus.80983

**Published:** 2025-03-22

**Authors:** Daisuke Katsura, Ayako Inatomi, Shinsuke Tokoro, Shunichiro Tsuji, Takashi Murakami

**Affiliations:** 1 Department of Obstetrics and Gynecology, Shiga University of Medical Science, Otsu, JPN

**Keywords:** closure, constriction, ductus arteriosus diastolic velocity, ductus venosus pulsatility index, reopening of ductus arteriosus, right ventricular myocardial performance index, the ductus arteriosus

## Abstract

Although premature constriction or closure of the ductus arteriosus (PCDA) is associated with poor prognosis and early delivery is considered before deterioration occurs, some cases may improve and have a good prognosis, but the changes in fetal Doppler during the reopening of the ductus arteriosus are unclear, as are the factors related to its reopening. We encountered two cases of PCDA. In the first case, right cardiac function and ductus venosus flow normalized in a few days and the ductus arteriosus reopened spontaneously, and became vaginal delivery at 37 weeks of gestation. In the second case, labor induction was performed due to confirmed fetal cardiac stress associated with the closure of the ductus arteriosus at 38 weeks of gestation, and cesarean section was performed due to non-reassuring fetal status. The improvement in the right ventricular myocardial performance index, ductus venosus pulsatility index, the tendency for ductus arteriosus diastolic velocity to decrease, and confirmation of prograde flow into the pulmonary artery within a few days could serve as predictive indicators for the reopening of the ductus arteriosus.

## Introduction

Premature constriction or closure of the ductus arteriosus (PCDA) is a condition in which the ductus arteriosus constricts due to various factors such as non-steroidal anti-inflammatory drugs (NSAIDs), polyphenols, or without any identifiable cause. This leads to increased fetal pulmonary blood flow, resulting in right heart failure and fetal hydrops [1-5]. The diagnosis of PCDA is established regardless of gestational age when the diameter of the ductus arteriosus is narrowed, with a systolic velocity > 1.4 m/s, a persistent diastolic velocity > 0.35 m/s, and a ductus arteriosus pulsatility index (PI) < 1.9, or complete closure of the ductus arteriosus due to the absence of ductal flow regardless of gestational age [5,6]. 

In severe cases, PCDA can result in intrauterine fetal death, postnatal circulatory failure, and persistent pulmonary hypertension. After birth, the immediate decrease in pulmonary vascular resistance with lung expansion and oxygenation alleviates the severe right ventricular afterload imposed by the constricted ductus. As a result, the neonate is no longer dependent on the ductus arteriosus. Consequently, early delivery has traditionally been indicated to prevent fetal condition deterioration due to PCDA, thereby reducing the need for interventions [3,7]; however, it is well known that in NSAID- or polyphenol-induced PCDA, discontinuation results in the reopening of the ductus arteriosus [1,2], and even idiopathic cases may resolve spontaneously in up to 38% of cases [4]. In cases of PCDA, if the ductus arteriosus reopens, the subsequent prognosis is generally favorable [7].

Cases that improve can avoid premature birth and cesarean section and have a good prognosis, while those that do not resolve require early delivery [4]. Therefore, identifying the possibility of reopening is helpful for prenatal management. Understanding the course of the reopening of the ductus arteriosus is essential for effective monitoring, although this remains unclear.

In this report, we present two cases of PCDA, discuss their ultrasonographic findings, and explore the course of progression. We also propose management strategies for PCDA.

## Case presentation

Case 1

**Figure 1:**
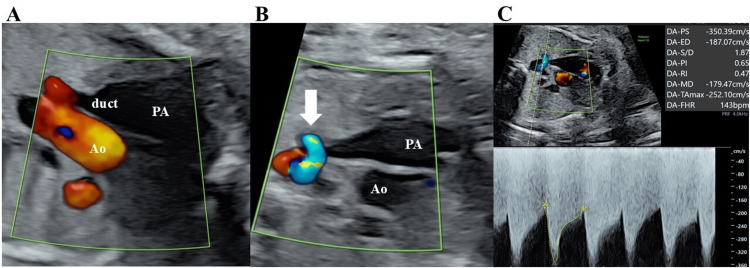
Fetal echocardiographic findings on the day of presentation in Case 1 (A) Enlargement of the ductus arteriosus and pulmonary artery with reduced prograde flow; (B) Narrowing and tortuosity of the ductus arteriosus and acceleration of ductal blood flow velocity (white arrow); (C) Doppler of ductal blood flow velocity (systolic velocity of 3.5 m/second and diastolic velocity of 1.87 m/second) and ductus arteriosus pulsatility index of 0.65. PA, pulmonary artery; Ao, aorta; duct, ductus arteriosus.

A 20-year-old woman (gravida 1, para 0) was referred to our hospital at 33 weeks and two days of gestation with suspicion of PCDA. Fetal ultrasound revealed an amniotic fluid volume of 17.3 cm and an estimated fetal weight (EFW) of 1994 g; severe tricuspid regurgitation (TR), enlargement of the pulmonary artery, narrowing and tortuosity of the ductus arteriosus diameter of 2.4 mm, prograde flow into the pulmonary artery diminishes, acceleration of ductal blood flow velocity (systolic velocity of 3.5 m/sec and diastolic velocity of 1.87 m/sec) and a decrease in ductus arteriosus pulsatility index (PI) of 0.65 were detected (Figure 1).

Therefore, constriction of ductus arteriosus was diagnosed. At this point, the fetal Doppler showed the following results: umbilical artery (UA) PI of 0.87, middle cerebral artery (MCA) PI of 1.36, ductus venosus (DV) PI of 0.87, inferior vena cava preload index (PLI) of 0.32, cardiothoracic area ratio (CTAR) of 26.8%, ratio of mitral valve diameter to tricuspid valve diameter (MV/TV) of 0.87, and myocardial performance index (MPI) index (left/right ventricle) of 0.27/0.72. No umbilical vein (UV) pulsation or fetal hydrops were detected. No obvious causative medications were identified. She was managed with hospitalization for close monitoring. We decided to closely monitor the fetal condition to prevent preterm birth, as the fetal heart rate monitoring indicated reassuring fetal status. The Doppler findings observed over the course of the next few days are shown in Table 1.

**Table 1:** Doppler findings observed from the initial visit onward in Case 1 w, week; d, day; DA, ductus arteriosus; PI, pulsatility index; DV, ductus venosus; Rt-MPI, right ventricular myocardial performance index; TR, tricuspid regurgitation

Gestational age	33w2d	33w3d	33w4d	33w5d	33w6d	34w0d	34w1d	34w2d	34w3d	34w4d
DA systolic velocity (m/s)	3.50	2.90	2.51	3.02	2.51	1.09	2.67	2.06	1.71	2.03
DA diastolic velocity (m/s)	1.87	0.88	0.66	1.63	0.84	0.57	0.39	0.26	0.09	0.02
DAPI	0.65	1.12	1.23	0.60	1.82	1.16	1.66	2.23	2.54	3.13
DVPI	0.87	0.94	0.42	0.51	0.21	0.31	0.38	0.59	0.27	0.48
Rt-MPI	0.72	0.48	0.33	0.32	0.43	0.56	0.27	0.54	0.30	0.19
TR	severe	moderate	moderate	moderate	moderate	mild	mild	mild	mild	mild

**Figure 2:**
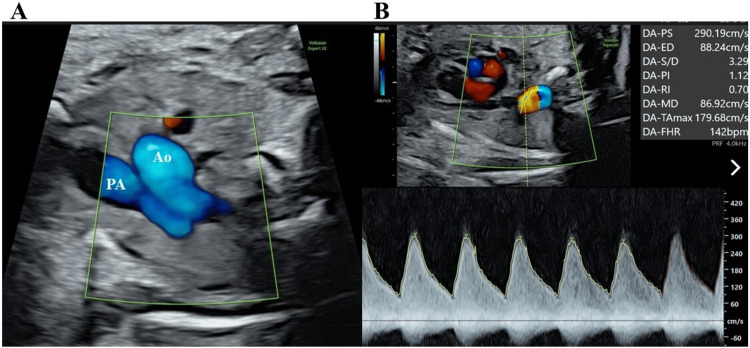
Fetal echocardiographic findings on the second day in Case 1 (A) The prograde flow into the pulmonary artery; (B) Doppler of ductal blood flow velocity (systolic velocity of 2.9 m/second and diastolic velocity of 0.88 m/second) and ductus arteriosus pulsatility index of 1.12. PA, pulmonary artery; Ao, aorta

Since the right heart stress tended to improve and the prograde flow into the pulmonary artery normalized on the second day of hospitalization (Figure 2), we predicted the reopening of the ductus arteriosus.

**Figure 3:**
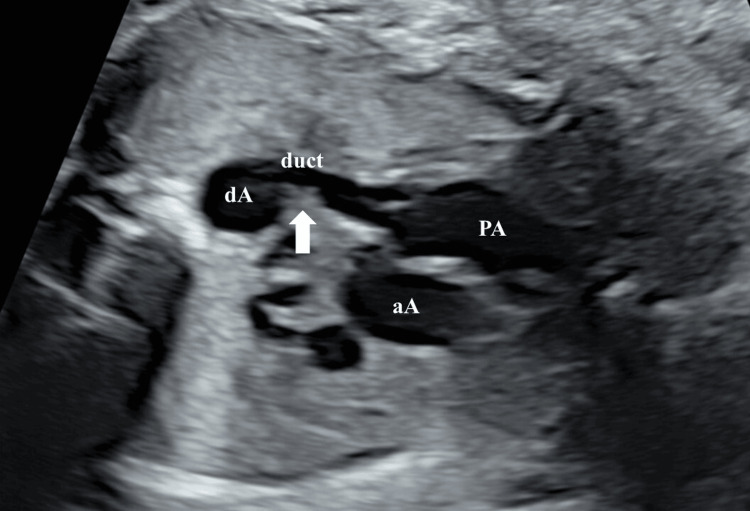
Fetal echocardiographic findings in Case 1 showing ridge of the site of stenosis and tortuosity (white arrow) PA, pulmonary artery; aA, ascending aorta; dA, descending aorta; duct, ductus arteriosus.

The reopening was confirmed, and a ridge was observed at the site of the previous stenosis, with tortuosity also confirmed (Figure 3).

**Figure 4:**
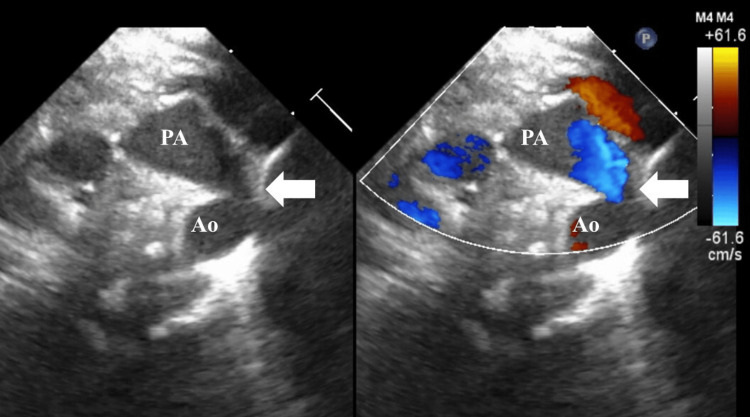
Postnatal echocardiograph in Case 1 showing ridge and tortuosity of the ductus arteriosus (white arrow) PA, pulmonary artery; Ao, aorta

Considering the improvement in fetal Doppler findings, she was discharged at 35 weeks of gestation after being informed about the potential triggers of PCDA. A vaginal delivery occurred at 37 weeks and four days of pregnancy. A male infant weighing 2416 g was delivered, with Apgar scores of 9 and 10 at one and five minutes, respectively, and umbilical artery pH of 7.279. The postnatal echocardiogram revealed a ridge and tortuosity of the ductus arteriosus (Figure 4); however, on the third day of life, the ductus arteriosus had closed spontaneously. The baby did not require oxygen and the course was stable.

Case 2

**Figure 5:**
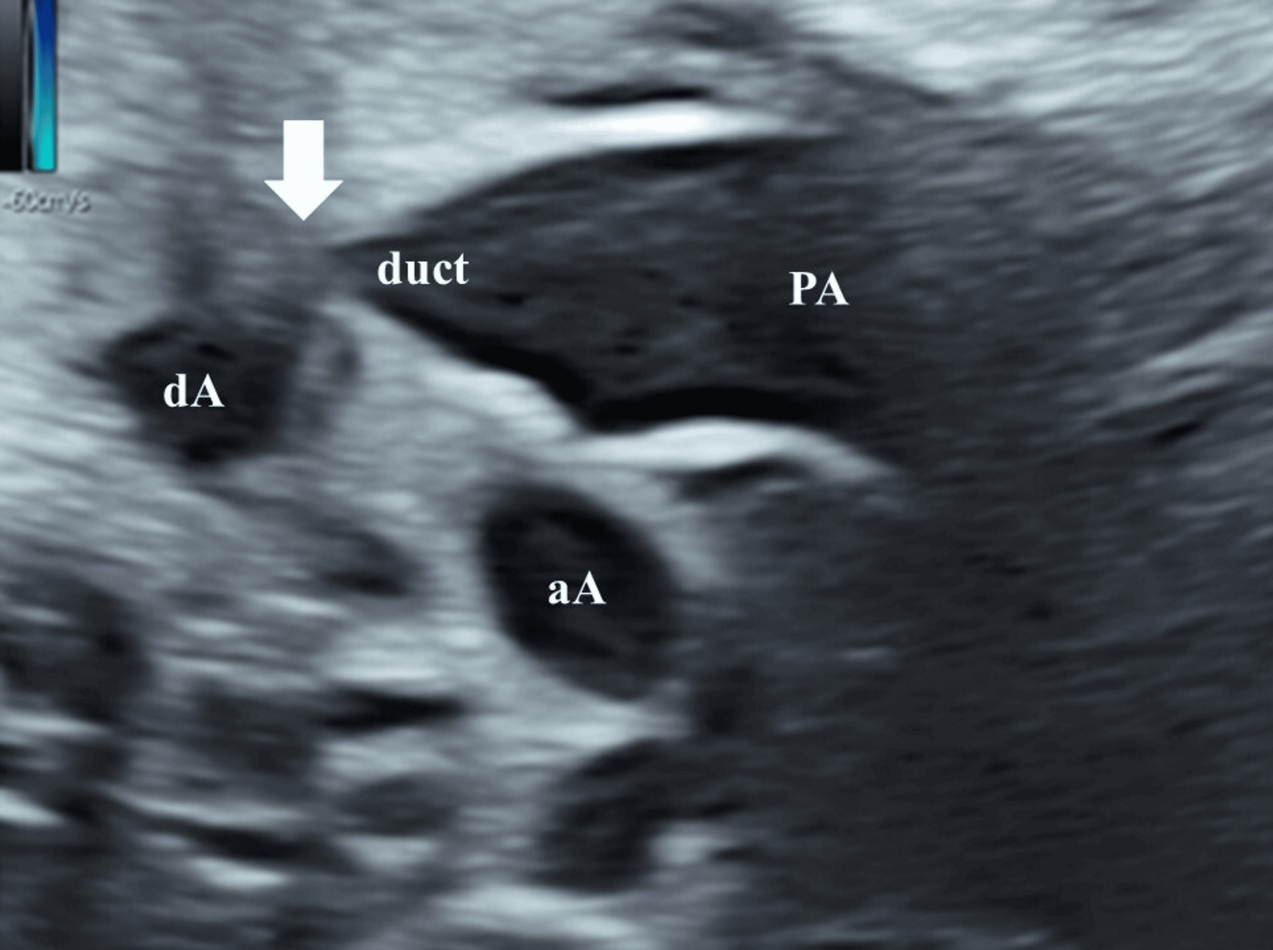
Fetal echocardiogram in Case 2 showing enlargement of the ductus arteriosus and pulmonary artery, closure of the ductus arteriosus (white arrow) aA, ascending aorta; dA, descending aorta; duct, ductus arteriosus.

A 33-year-old woman (gravida 3, para 2) was referred to our hospital at 38 weeks and one day of gestation with suspicion of PCDA. Fetal ultrasound revealed an amniotic fluid volume of 10 cm and EFW of 3059 g; right-sided heart enlargement (MV/TV 0.56), severe TR, pulmonary valve regurgitation (PR), enlargement of the pulmonary artery, and absence of ductal blood flow were detected (Figure 5).

**Figure 6:**
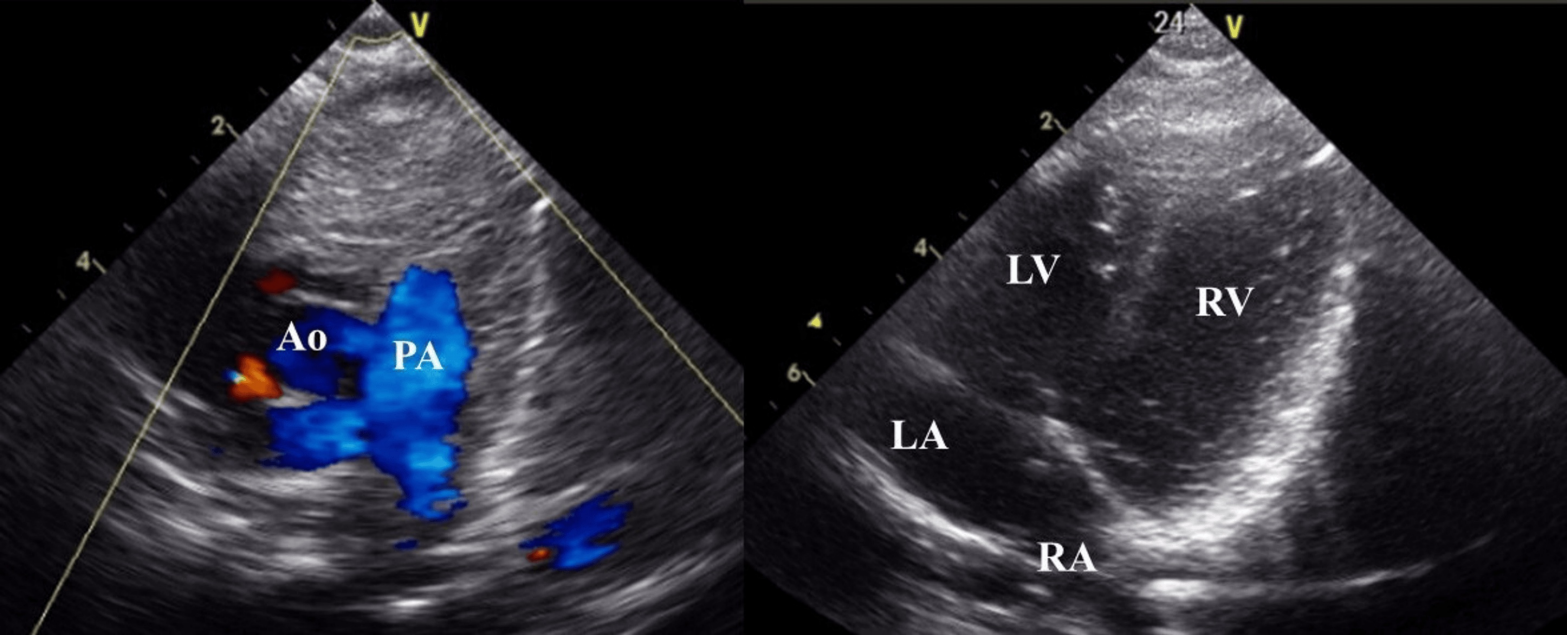
Postnatal echocardiographic findings in Case 2 showing no ductus arteriosus due to closure of the ductus arteriosus, and mild dilatation and hypertrophy of the right ventricle PA, pulmonary artery; Ao, aorta; LA, left atrium; LV, left ventricle; RA, right atrium; RV, right ventricle.

Therefore, the closure of ductus arteriosus was diagnosed. At this point, the fetal Doppler showed the following results: UA-PI of 0.63; MCA-PI of 1.93, DV-PI of 1.23; CTAR of 29.2%, inferior vena cava PLI of 0.32, MPI index (left/right ventricle) of 0.29/0.70. No UV pulsation or fetal hydrops were detected. Labor induction was initiated due to fetal cardiac stress, as the pregnancy had reached full term. Subsequently, although the fetal heart rate remained within the normal range, a decrease in baseline variability and recurrent late decelerations associated with uterine contractions prompted the decision to perform a cesarean section due to non-reassuring fetal status (NRFS). A female infant weighing 3018 g was delivered, with Apgar scores of 8 and 9 at one and five minutes, respectively, and umbilical artery pH of 7.338. The baby required supplemental oxygen for five days but did not need mechanical ventilation. Although the postnatal echocardiogram confirmed the closure of the ductus arteriosus and the mild dilatation and hypertrophy of the right ventricle (Figure 6), they improved with oxygen administration.

The baby's condition was stable, and no further tests were conducted. The baby was discharged after eight days, with no clinical complications.

## Discussion

PCDA may be associated with poor fetal outcomes, such as intrauterine fetal death, when the diagnosis is delayed [3,7]. Therefore, early prenatal diagnosis is crucial. The prevailing belief is that when signs of right heart failure with hydrops or NRFS, such as a decrease or absence in baseline variability and recurrent late decelerations, on fetal heart rate monitoring are present, pregnancy should be terminated promptly [8]. Some cases may improve and have a good prognosis [2-4]; however, excessive deterioration can be detrimental at both fetal and neonatal stages [3,7]. 

Early assessment of whether the condition will improve is crucial. Ultrasound findings serve as an important diagnostic tool in this evaluation. In cases of PCDA, the blood flow velocity in the ductus arteriosus accelerates, and the PI decreases [5,6]. In cases of deterioration and closure, blood flow becomes undetectable. Due to the volume load, right-sided cardiac failure can develop, leading to right ventricular and right atrial enlargement, TR, PR, increased preload indicators such as an elevated PLI and DV-PI, and impaired right ventricular function such as elevated right ventricular MPI (Rt-MPI) [3-5,9]. 

In Case 1 with the reopening of the ductus arteriosus, no PR was observed. Along with the acceleration of ductal blood flow, severe TR, a decrease in the ductus arteriosus PI, an elevation in DV-PI, and an increase in Rt-MPI were noted. The sequence of normalization observed was as follows: Rt-MPI → DV-PI → ductus arteriosus PI → ductus arteriosus diastolic velocity. Severe TR improved to moderate TR as the Rt-MPI normalized. As the ductus arteriosus PI and ductus arteriosus diastolic velocity normalized, moderate TR further improved to mild TR. The Rt-MPI normalized on the second day of hospitalization, while the DV-PI showed improvement on the third day. The ductus arteriosus diastolic velocity began to decrease on the second day and normalized on the eighth day. Although the ductus arteriosus systolic velocity did not completely normalize, a gradual decline was observed in the following days. In addition, the prograde flow into the pulmonary artery was confirmed on the second day. It has been reported that if the underlying cause of PCDA is removed, cardiac function normalizes within 24 hours to a few days, the ductus arteriosus reopens within four to seven days, and in cases of PCDA where the ductus arteriosus diastolic velocity was low and the ductus arteriosus PI tended to be high, the ductus arteriosus was more likely to be reopened [1,4]. 

Furthermore, considering the progression of the pathology of PCDA, the sequence of improvement in ultrasound findings and the time course of these improvements observed in our cases appeared to be reasonable. Therefore, the improvement in Rt-MPI and DV-PI, along with a tendency for a decrease in ductus arteriosus diastolic velocity and confirmation of prograde flow into the pulmonary artery within a few days, could serve as predictive indicators for the reopening of the ductus arteriosus. Based on these findings, it can determined that no intervention, such as labor induction, is necessary. Among these findings, the ductus arteriosus diastolic velocity, which directly reflects the condition of the ductus arteriosus, may be particularly useful.

In Case 1, following the reopening of the ductus arteriosus, a ridge, and tortuosity were observed, with the ridge likely indicating the site of the narrowing. Postnatally, the ridge and tortuosity of the ductus arteriosus were also confirmed and subsequently resolved with the spontaneous closure of the ductus arteriosus. The ridge was considered a reversible pathology associated with stenosis. Regarding tortuosity, it has been reported that there is a tendency for increased curvature as pregnancy progresses [9], and it is generally believed that there are no concerns if the ductus arteriosus closes spontaneously.

In Case 2 with closure of the ductus arteriosus, no ductal blood flow was observed. PR, severe TR, an increase in Rt-MPI, and an elevation in DV-PI were noted. Labor induction was initiated; however, the fetus developed NRFS and the fetal heart was under sufficient load to induce not only an increase in Rt-MPI but also PR. It has been reported that uterine contractions impose a load on the fetal venous Doppler and body cavity pressure [10]. Although uterine contractions result in a 60% reduction in uteroplacental perfusion, leading to transient fetal and placental hypoxia, a normal fetus can accommodate this hypoxia by enabling the fetal heart to respond to peripheral chemoreflexes, thereby maintaining cardiac output [11]. We speculated that the additional load from uterine contractions may exacerbate cardiac stress and that when the cardiac stress exceeds the fetus’s ability to adapt to the hypoxia induced by uterine contractions, it may potentially lead to NRFS. Reducing cardiac load by reopening the ductus arteriosus is expected to reduce NRFS and cesarean section. In Case 1, hospitalization for close monitoring was chosen due to the absence of hydrops and the reassuring fetal status on fetal heart rate monitoring. In contrast, labor induction was performed in Case 2 due to fetal cardiac stress. However, in retrospect, we might have preferred to manage Case 2 with close monitoring as well, given the absence of hydrops and the reassuring fetal status observed on fetal heart rate monitoring.

In Case 1, the diagnosis was constriction of the ductus arteriosus, while in Case 2, the ductus arteriosus was closed. Although there were differences in the ultrasound findings between the two cases, the mother in both cases did not experience any symptoms, such as decreased fetal movements. If the fetal condition worsens, decreased fetal movements may be detected [3,7,12]; however, it is crucial to make a diagnosis before the fetal condition deteriorates and maternal symptoms develop. Therefore, ultrasound markers for early detection are needed. In Case 2, right-sided heart enlargement was observed, while no enlargement of the right heart was noted in Case 1. However, severe TR was present in both cases. Right-sided heart enlargement is noted in 65% of cases of PCDA, while TR is observed in 90% of cases [1]. Therefore, evaluating right-sided heart enlargement alone is insufficient for diagnosing PCDA, and screening for TR using color Doppler is essential. Although ultrasonographic findings of the ductus arteriosus and pulmonary artery and regurgitation are useful for diagnosing PCDA [1], they are not commonly assessed during routine pregnancy checkups. Right-sided heart enlargement and TR are recommended as screening for PCDA [1]. Screening is recommended from the 24th week of gestation considering the sensitivity of the ductus arteriosus [13,14]. Regarding right-sided heart enlargement, a ratio of mitral valve diameter to tricuspid valve diameter < 0.8 is an indicator [15]. Regarding TR, mild TR, in which both the end of the regurgitation beam is located at the level of the tricuspid valve tip and the area of the regurgitation beam accounts for less than 30% of the right atrial area, is an indicator because the prevalence of mild fetal TR among normally grown fetuses is low at 6.23% [16] and 4.74% [17], and disappeared around 29 weeks of gestation [17]. However, it must be noted that TR may be associated with various fetal conditions other than PCDA [15,16].

To clarify the usefulness of this management approach, further accumulation of observational data regarding changes in ultrasonographic findings of MPI, DV-PI, ductus arteriosus PI and velocity, TR, and PR in cases of PCDA is necessary. However, with reference to previous reports [1-7], this approach shows a potential for being beneficial.

## Conclusions

if right-sided heart enlargement and/or mild or greater TR are detected in screening from the 24th week of gestation during routine pregnancy checkups, PCDA should be considered. Once PCDA is diagnosed, it is important to inquire about the use of NSAIDs and polyphenols, which are associated with PCDA, and hospitalization is required for removal of the causative agent and close monitoring. Fetal echocardiography should be performed every day until confirming the reopening of the ductus arteriosus as close monitoring. If fetal hydrops is absent and there is no immediate need for urgent delivery based on fetal heart rate monitoring, the progression of Rt-MPI, DV-PI and ductus arteriosus diastolic velocity and presence or absence of prograde flow into the pulmonary artery should be monitored to assess the possibility of ductal recanalization. If these findings do not improve within a few days and instead worsen, accompanied by at least one of pericardial, ascites, or pleural effusion, delivery should be considered. To the best of our knowledge, this is the first case report identifying predictive indicators using ultrasonography for the reopening of the ductus arteriosus in cases of PCDA.

## References

[1] Luchese S, Mânica JL, Zielinsky P (2003). Intrauterine ductus arteriosus constriction: analysis of a historic cohort of 20 cases. Arq Bras Cardiol.

[2] Zielinsky P, Piccoli AL Jr, Vian I (2013). Maternal restriction of polyphenols and fetal ductal dynamics in normal pregnancy: an open clinical trial. Arq Bras Cardiol.

[3] Soslow JH, Friedberg MK, Silverman NH (2008). Idiopathic premature closure of the ductus arteriosus: an indication for early delivery. Echocardiography.

[4] Aoki H, Kawataki M, Kim K (2023). Reopening of ductus arteriosus in idiopathic premature constriction or closure of ductus arteriosus: a case series. J Neonatal Perinatal Med.

[5] Wen J, Guo X, Cai S, Xu D, Zhang G, Bai X (2022). Fetal ductus arteriosus premature constriction. Int Heart J.

[6] Enzensberger C, Wienhard J, Weichert J, Kawecki A, Degenhardt J, Vogel M, Axt-Fliedner R (2012). Idiopathic constriction of the fetal ductus arteriosus: three cases and review of the literature. J Ultrasound Med.

[7] Lopes LM, Carrilho MC, Francisco RP, Lopes MA, Krebs VL, Zugaib M (2016). Fetal ductus arteriosus constriction and closure: analysis of the causes and perinatal outcome related to 45 consecutive cases. J Matern Fetal Neonatal Med.

[8] Genovese F, Marilli I, Benintende G (2015). Diagnosis and management of fetal ductus arteriosus constriction-closure. J Neonatal Perinatal Med.

[9] Benson CB, Brown DL, Doubilet PM, DiSalvo DN, Laing FC, Frates MC (1995). Increasing curvature of the normal fetal ductus arteriosus with advancing gestational age. Ultrasound Obstet Gynecol.

[10] Katsura D, Takahashi Y, Iwagaki S (2018). Changes in intra-amniotic, fetal intrathoracic, and intraperitoneal pressures with uterine contraction: a report of three cases. Case Rep Obstet Gynecol.

[11] Turner JM, Mitchell MD, Kumar SS (2020). The physiology of intrapartum fetal compromise at term. Am J Obstet Gynecol.

[12] Efkarpidis S, Alexopoulos E, Kean L, Liu D, Fay T (2004). Case-control study of factors associated with intrauterine fetal deaths. MedGenMed.

[13] Moise KJ Jr (1993168). Effect of advancing gestational age on the frequency of fetal ductal constriction in association with maternal indomethacin use. Am J Obstet Gynecol.

[14] Vermillion ST, Scardo JA, Lashus AG, Wiles HB (1997177). The effect of indomethacin tocolysis on fetal ductus arteriosus constriction with advancing gestational age. Am J Obstet Gynecol.

[15] Sharland GK, Allan LD (1992). Normal fetal cardiac measurements derived by cross-sectional echocardiography. Ultrasound Obstet Gynecol.

[16] Gembruch U, Smrcek JM (1997). The prevalence and clinical significance of tricuspid valve regurgitation in normally grown fetuses and those with intrauterine growth retardation. Ultrasound Obstet Gynecol.

[17] Clerici G, Romanelli M, Tosto V, Tsibizova V, Di Renzo GC (2021). Fetal transient tricuspid valve regurgitation: sonographic features and clinical evolution. J Matern Fetal Neonatal Med.

